# A Modern View of the Interstitial Space in Health and Disease

**DOI:** 10.3389/fvets.2020.609583

**Published:** 2020-11-05

**Authors:** Randolph H. Stewart

**Affiliations:** Department of Veterinary Physiology and Pharmacology, Michael E. DeBakey Institute, Texas A&M University, College Station, TX, United States

**Keywords:** interstitial pressure, extracellular matrix, lymphatic system, edema, shock, inflammation, sodium balance

## Abstract

Increases in the volume of the interstitial space are readily recognized clinically as interstitial edema formation in the loose connective tissue of skin, mucosa, and lung. However, the contents and the hydrostatic pressure of this interstitial fluid can be very difficult to determine even in experimental settings. These difficulties have long obscured what we are beginning to appreciate is a dynamic milieu that is subject to both intrinsic and extrinsic regulation. This review examines current concepts regarding regulation of interstitial volume, pressure, and flow and utilizes that background to address three major topics of interest that impact IV fluid administration. The first of these started with the discovery that excess dietary salt can be stored non-osmotically in the interstitial space with minimal impact on vascular volume and pressures. This led to the hypothesis that, along with the kidney, the interstitial space plays an active role in the long-term regulation of blood pressure. Second, it now appears that hypovolemic shock leads to systemic inflammatory response syndrome principally through the entry of digestive enzymes into the intestinal interstitial space and the subsequent progression of enzymes and inflammatory agents through the mesenteric lymphatic system to the general circulation. Lastly, current evidence strongly supports the non-intuitive view that the primary factor leading to inflammatory edema formation is a decrease in interstitial hydrostatic pressure that dramatically increases microvascular filtration.

## Introduction

The traditional view that the interstitial space is a relatively static and, frankly, uninteresting region is being reassessed in light of new research showing that the interstitium plays an active role in the regulation of interstitial volume and content and is a key participant in the pathogenesis of inflammation and shock. The passive view holds that changes in interstitial volume and pressure are the result of influences outside of the interstitium, i.e., microvascular pressure, microvascular permeability or plasma colloid osmotic pressure. We now know, however, that the generally predictable relationship between interstitial volume and interstitial pressure can change markedly and that these transitional episodes can occur within minutes. On another front, recent investigations have revealed that the interstitial space plays an active role, in addition to the kidney, in the regulation of salt and water balance in the body including blood pressure. A third avenue of inquiry has confirmed that the intestinal interstitial space and mesenteric lymphatic system provide a critical link between circulatory shock and the subsequent systemic inflammatory response syndrome. This review examines current concepts regarding regulation of interstitial volume, pressure and flow and, utilizing that background, addresses topics of interest that impact IV fluid administration.

## The Interstitium

The interstitial space that lies between blood vessels and cells provides the fluid and structural environment surrounding those cells. Under most conditions in most tissues, fluid from the vascular space continually filters from the microvessels into the interstitial space and is not reabsorbed (1). Notable exceptions include the peritubular capillaries in the kidney and microvascular beds within intestinal villi that routinely absorb interstitial fluid (2, 3). Interstitial fluid is removed *via* lymphatic drainage and returned to the venous circulation. In organs located in the pleural, pericardial and peritoneal spaces, some interstitial fluid filters through the organ's serosal surface into the surrounding fluid space and, then, is also taken up into the lymphatic system. In the edematous intestine, interstitial fluid can cross the mucosal barrier into the lumen (4, 5). All of these fluid flows–microvascular filtration, lymph flow, and trans-serosal flow–are significantly influenced by interstitial fluid (hydrostatic) pressure. An increase in interstitial fluid pressure leads to a decrease in microvascular filtration and to increases in lymph flow and trans-serosal flow. Therefore, it is the interplay between all of the factors affecting these flows (i.e., microvascular pressure, lymphatic contractility, serosal permeability, etc.) that determines the steady-state interstitial fluid pressure (6). The interstitial volume is then jointly determined by the interstitial pressure and the interstitial pressure-volume relationship (6).

The structural elements of the interstitial space, collectively called the extracellular matrix, primarily consist of types I and III collagen fibers, elastic fibers, microfibrils, and glycosaminoglycans (GAGs) (7). The GAGs are sulfated (heparin/heparan sulfate, chondroitin/dermatan sulfate and keratan sulfate) and non-sulphated (hyaluronan) (8, 9). The sulfated GAGs are covalently bound to a protein backbone creating a macromolecule called a proteoglycan. These sulfated GAGs carry a net negative charge and are, thus, capable of attracting, and binding cations, such as sodium ions (8, 10).

These interstitial structural elements make distinct mechanical contributions to the relationship between interstitial volume and interstitial fluid pressure. When fibroblasts are cultured *in vitro* in a collagen gel, the fibroblasts attach to the collagen fibers and, by exerting tension on those attachments, reduce gel volume (11). This fibroblast-mediated gel compaction is augmented *in vitro* by platelet-derived growth factor and inhibited by the inflammatory mediators, IL-1α, and PGE_2_ (12–14). A comparable effect is seen in the extracellular matrix of loose connective tissue found throughout the body, where fibroblasts attach to multiple collagen fibers *via* integrin connections and compact the matrix (15–18). This action, in concert with the microfibril network, acts to reduce interstitial volume and increase interstitial pressure (19). Conversely, GAGs, particularly hyaluronan, create an imbibition pressure similar to a sponge that acts to expand interstitial volume and decrease interstitial fluid pressure (19, 20). The interstitial fluid pressure-volume relationship and interstitial compliance, i.e., the slope of the interstitial pressure-volume relationship, in part, reflect the interaction between these two counterbalancing mechanical forces (7, 19, 21).

The normal interstitial fluid pressure in tissues such as skin, intestine and lung is subatmospheric on the order of −1 to −4 mmHg; while in other tissues, including liver, kidney, and myocardium, it is normally greater than atmospheric pressure (21–24). In all organs, however, an increase in interstitial volume following increased microvascular pressure and, thus, microvascular filtration is accompanied by an increase in interstitial pressure. This characterizes the normal interstitial pressure-volume relationship and interstitial compliance for each tissue (21, 25).

Protein molecules that filter from the microvasculature into the interstitial space are responsible for the colloid osmotic pressure (COP) exerted by the interstitial fluid. The interstitial protein concentration is determined, in part, by the protein permeability of the microvessels. Due to the high permeability of the hepatic sinusoids, the interstitial protein concentration in the liver is very similar to that of plasma; whereas, the low permeability of the blood-brain barrier ensures a very low protein concentration in the cerebrospinal fluid (26, 27). Interstitial protein concentration is also affected by the microvascular filtration rate. Because of the microvascular barrier's differential permeability to water and protein, an increase in the microvascular filtration rate leads to a fall in the protein concentration of the filtrate, the interstitial fluid and the lymph—a phenomenon called protein washdown (1, 28). Similarly, a decrease in filtration rate results in an increase in interstitial fluid and lymph protein concentrations (28, 29). Once protein has entered the interstitial space, it is removed and returned to the general circulation only by the lymphatic system.

**Figure 1:**
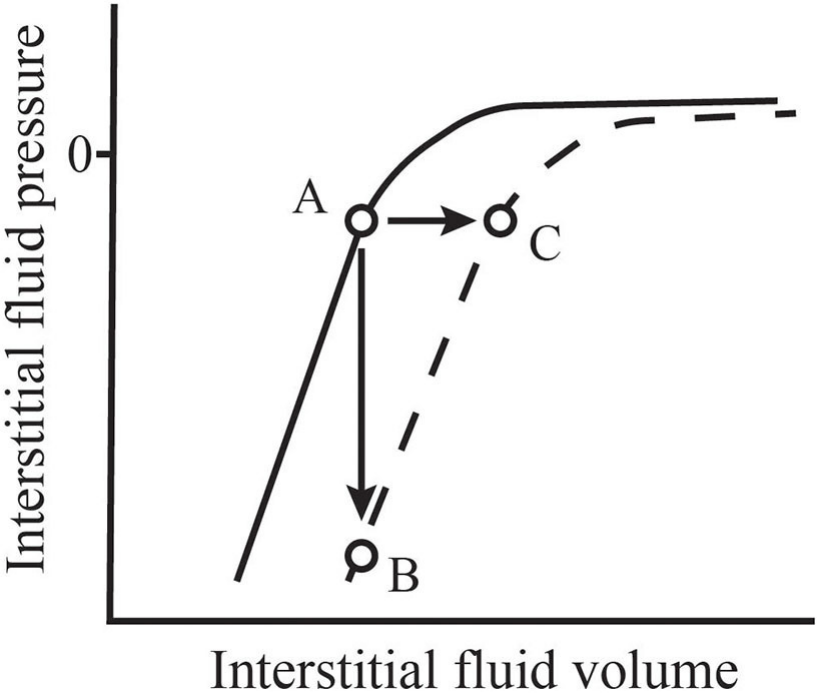
Normal values for interstitial fluid volume and pressure (A) are located along the normal interstitial pressure-volume relationship (solid line). During inflammation, this relationship shifts (dashed line). If microvascular filtration is impeded during this process, thus limiting an increase in volume, interstitial pressure falls precipitously (B). However, if filtration is allowed to continue, the filtration rate may increase 10–20-fold and interstitial volume expands rapidly with little change in pressure (C). Adapted from Aukland and Reed (21) and Stewart (30).

Interstitial compliance in a given tissue is not constant, but rather is a function of interstitial volume. The solid line in Figure 1 represents the general shape of the interstitial pressure-volume relationship that occurs in tissues like skin and muscle (21). In that figure, we see that, at normal to low volumes, the interstitium has a very low compliance where a small change in volume results in a large change in interstitial fluid pressure (21). At higher volumes, compliance increases allowing interstitial volume to expand with only small increases in pressure. The normal low volume-low compliance state is relevant in understanding the role of interstitial fluid in hemorrhage. In his seminal paper in 1896, Starling commented that it was already well-known that, within a short period following hemorrhage, the blood “contains less hemoglobin and blood corpuscles and relatively more plasma” (31). He concluded that this phenomenon occurs because fluid is absorbed from tissues by the blood vessels. Numerous studies since have confirmed the existence of this hemorrhage-induced shift of interstitial fluid into the vascular space that results from a fall in capillary hydrostatic pressure—a process now called transcapillary refill (32–35). Transcapillary refill is transient however, lasting ~1 h (32, 35). It is limited by low interstitial compliance because, as fluid is reabsorbed from the interstitial space, interstitial fluid pressure falls. In addition, reabsorption of interstitial fluid into microvessels reverses the protein washdown effect and increases interstitial protein concentration and COP (1). The combined effects of decreased interstitial fluid pressure and increased interstitial COP prevent further fluid reabsorption (1).

### The Interstitium in Salt and Water Balance

The conventional view of the regulation of blood volume and pressure has focused on the kidney's ability to regulate total body sodium content (36). More recent information has required an adjustment to that view. While the importance of the kidney in long-term pressure regulation remains unquestioned, it is now known that the interstitial space, particularly of the skin, can actively store sodium in non-osmotic form and, thus, plays an important role in total body sodium and water balance and long-term regulation of blood pressure (37). In 2000, it was reported in a study of human subjects that a high salt diet induced an increase in plasma volume and total body sodium, but no increase in extracellular fluid volume or body weight and no increase in arterial blood pressure (38). Subsequently, a long-term study of humans in simulated space flight that allowed tight control of sodium intake demonstrated no relationship between total body sodium ion and blood pressure (39).

In rats fed a high salt diet, Na^+^ storage in skin is increased without a concomitant increase in skin water storage indicating that the sodium is stored non-osmotically (10, 40). In addition, the increased skin sodium content correlated with an increase in the total skin GAG content suggesting that the dermal sodium is stored bound to the negatively charged GAGs within the interstitial space and that the skin GAG content increases adaptively in response to the high sodium load (10). Similarly, a recent study of human subjects demonstrated a positive correlation between the GAG content of skin, artery, and muscle tissue and tissue total sodium levels (41). It was subsequently demonstrated in rats that a high salt diet induced a change in the GAGs in skin toward greater sulfation and higher charge density (42). This change was characterized by an increase in the sulfated GAG-to-hyaluronan ratio. Comparable changes were observed in human heart failure patients that showed increased density of total GAGs and sulfated GAGs in the interstitium of skin biopsies (43). The more negative charge carried by sulfated GAG component of proteoglycans compared to hyaluronan enhances the ability of these matrix components to bind Na^+^ (44).

These findings indicate that an active regulatory mechanism responds to long-term high dietary salt by increasing the total GAG content of skin including a relative increase in the strongly negatively charged sulfated GAGs thus enhancing the ability of the skin to act a reservoir for non-osmotic storage of Na^+^. Interestingly, when challenged with a high-salt diet, rats prone to salt-sensitive hypertension show a reduced ability to store Na^+^ non-osmotically compared to control rats (45).

In addition to the changes in interstitial GAG content, high dietary salt in rats also leads to interstitial infiltration of immune cells of the mononuclear phagocyte system (MPS) including macrophages and to lymphatic capillary hyperplasia in skin with enhanced clearance of sodium and chloride (46, 47). The MPS cells appear to play a regulatory role in this process. High interstitial salt concentrations activate the tonicity-responsive enhancer-binding protein transcription factor in MPS cells leading to secretion of vascular endothelial growth factor-C, a promoter of lymphangiogenesis and lymphatic hyperplasia (46). The high salt diet also leads to a modest, but measurable, increase in arterial blood pressure. Depletion of MPS cells blocks this response to high salt intake and increases its hypertensive effect. A subsequent study demonstrated that pretreatment with an inhibitor of vascular endothelial growth factor exaggerated the hypertensive effect of a high salt diet in rats but did not inhibit the lymphatic hyperplasia in skin (48). Therefore, while the interstitial space of the skin clearly plays an important role in sodium and water balance and long-term regulation of blood pressure, the role of dermal lymphatic development in this regulatory process remains unclear.

## The Lymph System and Lymphatic Drainage

The lymphatic system drains interstitial fluid first through blind-end initial lymphatic capillaries located within the interstitial space (49). With diameters up to 100 μm, these lymphatic capillaries are larger than blood capillaries and contain no mural smooth muscle. This fluid, now called lymph, is transported to larger collecting lymphatic vessels that contain smooth muscle and regularly spaced unidirectional valves. Lymph continues to flow through progressively larger vessels such as the thoracic duct and right lymphatic duct and, then, empties into the blood circulation at the great veins of the neck.

Collecting lymphatic vessels contract spontaneously and are capable of driving flow against an axial pressure gradient. The pumping activity of these lymphatic vessels has been demonstrated to respond to mechanical stimuli and to numerous vasoactive mediators (50–53). In response to increased luminal pressure (hoop stress), these contractile lymphatics increase the strength and frequency of contraction (50). While, in response to increased luminal flow (shear stress), they dilate and decrease their contraction frequency (52).

Lymph flow through the intact lymphatic network has been modeled using the Drake-Laine equation

$$\begin{array}{l} {\text{J}}_{\text{L}}=\left({\text{P}}_{\text{int}}+{\text{P}}_{\text{p}}-\text{SVP}\right)/{\text{R}}_{\text{L}} \end{array}$$

in which J_L_ is lymph flow, P_int_ is the interstitial fluid pressure, P_p_ is the effective driving pressure contributed by the intrinsic pumping activity of lymphatic vessels and extrinsic compression, SVP is the systemic venous pressure to which the lymph is flowing and R_L_ is the effective resistance to flow offered by the lymphatic network (54). The driving pressure provided by lymphatic pumping explains why tissues such as skin and lung can have subatmospheric interstitial fluid pressure and why lymph flows from that low-pressure region to the higher-pressure veins. The “effective” lymphatic resistance (R_L_) in the Drake-Laine equation was derived from the slope of the flow-outflow pressure relationship in a multi-branched system of cyclically contracting lymphatic vessels (54). Therefore, rather than being a simple measure of the hydraulic resistance of a tube, it is one measure of the behavior of a complex system. This suggests that its value can be affected by many factors including lymphatic contractility and contraction rate as well as interactions between lymphatic vessels.

Increases in interstitial fluid pressure, which is the upstream pressure for the lymphatic system, have been shown to dramatically increase lymph flow following increases in microvascular filtration in the affected organ. In rat experiments, intravenous administration of 0.9% NaCl solution at a rate of 0.2 ml·min^−1^·100 g body wt^−1^ for 45 min resulted in a 25-fold increase in measured flow from the main intestinal lymph trunk (55). Interestingly, the increase in lymph flow calculated from dimensional changes in mesenteric lymphatic vessels and the contraction frequency was closer to a 2.5-fold increase. A likely explanation for this large discrepancy is that, under conditions of marked increases in P_int_, the resulting increase in lymph flow is the result of enhanced *passive* flow rather than increased lymphatic pumping. This is because, when upstream (interstitial) pressure exceeds downstream (venous) pressure, the passive flow generated outstrips the pumping capacity of the lymphatic vessel (56). In fact, under these conditions, stimulation of lymphatic contractile activity reduces, rather than augments, flow (57). This observation illustrates the counterintuitive physiologic utility of lymphatic vessel relaxation in response to increased shear stress since dilation and reduced pumping activity under high flow conditions will increase flow.

Venous pressure exerts two opposing effects on lymph flow. First, increased venous pressure leads to sequential increases in microvascular fluid pressure, microvascular filtration and interstitial pressure, thus enhancing lymph flow. Second, as seen in the Drake-Laine equation, systemic venous hypertension can reduce lymph flow by elevating lymphatic outflow pressure (58–60). In experimental studies, the effect of increasing lymphatic outflow pressure is exaggerated in anesthetized animals likely because the anesthetic agent reduces lymphatic contractility (61). In unanesthetized animals, elevation of systemic venous pressure has only a modest effect on lymph flow because the increase in lymphatic luminal pressure elicits a hoop stress-mediated increase in the strength and frequency of lymphatic pumping (60, 62). However, when it occurs in conjunction with any additional edemagenic challenge such as increased microvascular pressure, systemic venous hypertension significantly worsens edema formation (58).

The influence of lymphatic outflow pressure is also discernible in interactions within the lymphatic system. Lymph from both the intestine and the liver flows into the cisterna chyli prior to entering the thoracic duct. The capacity for the intestine to drive lymph flow against increased outflow pressure is less than that of the liver (63, 64). Therefore, increases in thoracic duct pressure induce a more pronounced reduction in mesenteric vs. hepatic lymph flow (63). In a canine model of caudal vena caval hypertension, increased lymph flow from the liver contributed to increased cisternal pressure and, thereby, reduced lymph flow from the intestine and exacerbated intestinal edema and ascites formation (64).

Lymphatic vessels not only respond acutely to changes in lymph flow and pressure, but also adapt chronically in response to mechanical stimuli. Vessels exposed to prolonged increases in luminal pressure respond within a few days by increasing pumping capacity (65). This adaptation carries the benefit of promoting active lymphatic pumping in response to a downstream obstruction or increase in outflow pressure. Conversely, a vessel exposed to high lymph flow due to increased microvascular filtration responds by becoming a weaker pump (66). This is also a beneficial change that would boost passive lymph flow in response to edema formation and increased interstitial fluid pressure.

## Intestinal Interstitium and Mesenteric Lymph in Trauma/Hemorrhagic Shock

Diminished blood flow to the gastrointestinal tract resulting from trauma/hemorrhagic shock plays a key role in the subsequent development of systemic inflammatory response syndrome (SIRS) including acute respiratory distress syndrome (ARDS) and multiple organ dysfunction syndrome (MODS) (67, 68). An early view that intestinal injury allowed entry of enteric bacteria and endotoxin into the intestinal interstitial space and, from there, into the mesenteric circulation was not well-supported by later studies (67–69).

An alternative hypothesis proposes that transit *via* the mesenteric lymphatic system allows proinflammatory agents to travel from the intestinal interstitial space to the general circulation bypassing the portal vein and liver. In 1970, Glenn and Lefer reported that diversion of thoracic duct lymph improved survival in a model of hemorrhagic shock (70). Later studies by Deitch et al. (71) focused on the intestinal interstitial space and its lymphatic drainage by demonstrating that while mesenteric lymph from a hemorrhagic shock model in rats was cytotoxic, portal vein plasma was not (71–73). In addition, when mesenteric lymph is prevented from entering the circulation, shock-induced injury to lung and heart is avoided and survival is enhanced (71, 74). Models of intestinal ischemia/reperfusion rather than hypovolemic shock showed similar results. Mesenteric lymph collected following intestinal hypoperfusion and, then, administered intravenously to clinically normal animals provokes tissue injury affecting the lungs and heart (75–77). Similarly, the myocardial edema induced in a canine model of mesenteric ischemia/reperfusion is eliminated by diversion of mesenteric lymph (75).

Mesenteric lymph also appears to play a central role in the pathogenesis of multiple organ failure associated with dermal burn injury (78). Ligation of the mesenteric lymphatic duct prevents cardiac dysfunction in rats with experimental burn injury (79). Interestingly, electrical stimulation of the vagus nerve increases intestinal tight junction proteins (i.e., occludins) in adjacent cells thereby protecting against intestinal barrier injury and peritoneal inflammatory response following dermal burn injury (80, 81).

Vagal nerve stimulation also increases intestinal blood flow, prevents intestinal barrier dysfunction, markedly decreases lung inflammation and inhibits development of inflammatory and cytotoxic activity in mesenteric lymph subsequent to hemorrhagic shock (82–86). As a therapeutic modality, vagal nerve stimulation is currently impractical. However, a pharmacologic vagal agonist, CPSI-121, also prevents intestinal injury and acute lung injury as well as attenuates development of inflammatory activity in mesenteric lymph following induction of hemorrhagic shock (87, 88).

The nature of the cytotoxic element in the affected mesenteric lymph has been addressed by several investigations focused on the entry of bacteria and endotoxin from the GI tract and activation of endogenous proinflammatory mediators (68). The hypothesis with the strongest current evidentiary support involves passage of pancreatic digestive enzymes through a damaged intestinal mucosa into the interstitial space. Mitsuoka et al. (89) demonstrated that luminal dilution and inhibition of digestive pancreatic enzymes diminished the lung injury induced by intestinal ischemia/reperfusion. Subsequently, they and others have provided evidence that intestinal hypoperfusion and ischemia/reperfusion injury lead to mucosal barrier disruption and entry of luminal components including free fatty acids and digestive enzymes from the exocrine pancreas into the intestinal interstitium (90–92). Once there, the pancreatic enzymes begin to digest interstitial components to produce novel inflammatory and cytotoxic agents such as unbound free fatty acids (93). These pancreatic enzymes and cytotoxic agents can then gain access to the general circulation *via* the mesenteric lymphatics as well as by the portal circulation and peritoneal cavity (71, 74, 94). Interruption of this pathologic cascade leads to more positive outcomes. Intraluminal administration of a protease inhibitor and a gastric and pancreatic lipase inhibitor reduces intestinal tissue injury and improves cardiovascular function (95). In addition, blockade of pancreatic enzymes within the bowel lumen increases survival in three forms of experimental shock caused by hemorrhage, septic peritonitis and intravenous endotoxin (96). Three different protease inhibitors instilled directly into the small intestine 1 h after initiation of the shock episode were all shown to have a beneficial effect (96).

## Serosal Transudation

Transudation or fluid filtration through the serosal covering of organs positioned within the pericardial, pleural and peritoneal spaces provides a second route for interstitial fluid removal in healthy organs. The factors governing filtration across the serosal barrier are approximated by a modified form of the Starling-Landis equation where interstitial fluid pressure acts to drive fluid flow out of the organ and interstitial COP acts to restrain that flow (97, 98). In both the heart and liver, increases in venous and microvascular pressures result in increases in interstitial fluid pressure and, thus, serosal transudation (97, 98). These findings are consistent with the clinical observations of pericardial effusion and ascites associated with pulmonary hypertension and right-sided heart disease (99–101).

In response to chronic edemagenic challenges, the permeability of the serosal surface can change over time. Following 5–6 weeks of caudal vena caval hypertension in dogs, the fluid and protein permeabilities of the hepatic serosal surface significantly decreased leading to significant decreases in serosal transudation and ascites volume (27).

## Edema: Causes, Mechanisms, and Consequences

Interstitial edema, the accumulation of excess fluid in the interstitial space, can lead to a number of negative consequences depending on the organ system involved. In addition to increasing oxygen diffusion distance within tissues, edema affecting the lung, heart and intestine impairs the organ's mechanical and physiological function (102–104). Interstitial edema in organs like the brain, intestines and kidney, where volume expansion is constrained, can lead to the development of compartment syndrome with resultant impairment of blood flow and organ failure (105). For the same reason, intestinal edema can also prevent surgical closure of an open abdomen. Pulmonary edema increases the work of breathing and carries the added risk of alveolar flooding.

Classically, interstitial edema forms as a result of some combination of increased microvascular pressure, decreased plasma COP, increased microvascular permeability and decreased lymphatic drainage. The effect of the first three of these factors is to increase the rate of microvascular filtration into the interstitial space as can be appreciated in the Starling-Landis equation (1). As discussed above, inhibition of lymphatic drainage caused by lymphatic obstruction or elevated lymphatic outflow pressure does not necessarily promote interstitial edema formation. However, it does magnify the impact of other edemagenic insults such as elevated microvascular pressure (58, 106).

Microvascular fluid pressure acts to promote microvascular filtration and is commonly increased in association with venous hypertension resulting from venous thrombosis or cardiac dysfunction. It is also increased by the arteriolar dilation that occurs in maldistributive shock associated with inflammation and sepsis. Plasma COP opposes microvascular fluid pressure and acts to restrain filtration, therefore decreased plasma COP due to hypoproteinemia/hypoalbuminemia also leads to greater filtration. This increase in filtration can have a broader impact than the easily recognized effects on lungs and skin. Hypoproteinemia in dogs has been shown to cause myocardial edema and impaired diastolic function (107). Intravenous administration of isotonic crystalloid solutions to normal subjects thus promotes interstitial edema formation by simultaneously increasing microvascular pressure *via* blood volume expansion and decreasing plasma colloid osmotic pressure *via* dilution.

Microvascular filtration can also be increased as a result of increases in the permeability of the microvascular barrier to either fluid or protein (1, 108–110). The permeability of the microvascular barrier to both is actively regulated at the level of the vascular endothelium and the glycocalyx layer located on the endothelial surface (111, 112). Numerous inflammatory mediators increase microvascular permeability leading to increased microvascular filtration and interstitial edema formation (111, 112).

### Inflammatory and Immune-Mediated Edema Formation

Changes in microvascular pressure and microvascular permeability are not the only and, perhaps, not even the most important causes of interstitial edema during inflammation. The standard view of microvascular filtration assumes that the interstitial fluid pressure is relatively stable and that it changes in a predictable fashion as interstitial volume increases and decreases. However, a series of experiments at the University of Bergen investigating inflammatory and immune-mediated alterations in the skin and tracheal mucosa of rats has revealed that interstitial pressure can fall precipitously thereby strongly promoting microvascular filtration and inducing a rapid increase in interstitial volume (7, 19).

Studies exploring these phenomena have taken one of two basic forms. Experiments that allow continued microvascular filtration following the inflammatory insult show an initial modest fall in interstitial fluid pressure followed by a return to near baseline values accompanied by a rapid increase in interstitial volume. In contrast, experiments that actively minimize microvascular filtration at the onset of the insult often show a profound fall in interstitial fluid pressure. These findings indicate a fundamental shift in the interstitial pressure-volume relationship (see Figure 1). Following dermal burn injuries in rats with continued filtration, intradermal interstitial fluid pressure fell from −1 to −31 mmHg within 15 min and, then, rose to approximately atmospheric pressure as edema developed (113). The same insult induced in rats immediately following euthanasia, thus limiting microvascular filtration, caused mean interstitial pressure to fall to −135 mmHg. Non-injured skin in these experiments showed no change in interstitial pressure.

Similar decreases in interstitial fluid pressure, although generally not as dramatic, have been induced in skin following local injection of numerous proinflammatory stimuli including PGE_1_, PGI_2_, histamine, cytochalasin D, xylene, carrageenan, TNF-α, IL-1β, and IL-6 (114–119). Ischemia-reperfusion injury and freezing injury have also been demonstrated to significantly lower interstitial fluid pressure and promote edema formation in skin (120, 121).

Edema formation associated with immune-mediated phenomena shows similar changes in interstitial fluid pressure. In a model of dextran anaphylaxis in rats, dermal interstitial pressure fell 5–10 mmHg in 20–40 min when circulatory arrest was induced 1 min following intravenous dextran administration (115, 122). In the absence of circulatory arrest, visible edema formed with no significant change in interstitial fluid pressure.

Inflammatory/immune-mediated challenges similar to those previously described also induce edema in tracheal mucosa associated with decreases in interstitial fluid pressure. Like skin, dextran anaphylaxis in rats was characterized by more negative interstitial pressure and rapid edema formation in the tracheal mucosa (122, 123). Tracheal interstitial pressure is also lowered by agents that induce mast cell degranulation (C48/80 and polymyxin B sulfate) as well as by stimulation of vagal nerve C fibers (124, 125).

Integrins, transmembrane proteins that facilitate fibroblast adhesion to the extracellular matrix, appear to play a central role in the inflammation-related fall in interstitial pressure (25). Subdermal injection of anti-β1 integrin IgG in rats with circulatory arrest caused a concentration-dependent decrease in interstitial fluid pressure of 4–6 mmHg in 10 min compared to preimmune IgG from the same source (126). In rats with intact circulation, anti-β1 integrin IgG caused interstitial volume to increase significantly within the same time frame. In those same studies, injection of anti-fibronectin IgG had no measurable effect on interstitial fluid pressure. In a subsequent study, intravenous administration of the anti-inflammatory agent, α-trinositol, had no effect on interstitial fluid pressure when used alone, but prevented the decrease in interstitial fluid pressure caused by subdermal administration of anti-β1 integrin IgG (127). Similarly, the ability of anti-α2β1 integrin IgG and anti-β1 integrin IgG to lower interstitial pressure in rat dermis was eliminated by simultaneous subdermal administration of platelet-derived growth factor-BB (PDGF-BB) (128). PDGF-BB exerts this effect by upregulating the expression of β3 integrin even though blockade of β3 integrin does not lower interstitial pressure (129). The investigators propose that normal tension within the extracellular matrix is maintained by β1 integrin-mediated contraction, that proinflammatory mediators disrupt the β1-integrin connections allowing rapid edema formation and that PDGF-BB re-establishes tension within the matrix and counteracts edema by stimulating the activity of β3-integrin (7).

In addition to its ability to counteract the effects of anti-β1 integrin IgG, pre-treatment with α-trinositol has been shown to eliminate or markedly attenuate the decrease in interstitial fluid pressure in skin and trachea in response to burn injury, freezing injury, subdermal injection of carrageenan and dextran anaphylaxis (120, 122, 130, 131). α-trinositol appears to have a modest ability to reduce edema formation when administered after, rather than before, the tissue insult. PDGF-BB, on the other hand, can normalize interstitial fluid pressure when administered 10–30 min following the insult (128, 129). In addition to α-trinositol and PDGF-BB, agents shown to prevent or reverse the fall in interstitial pressure include prostaglandin F_2_α, corticotropin releasing factor, insulin and vitamin C (117, 132–134).

This inflammatory/immune-mediated decrease in interstitial fluid pressure has a dramatic effect on microvascular filtration. Reed and Rodt calculated that appearance of visible edema within 10–20 min of the insult characteristic of inflammatory processes indicated a 50–100-fold increase in microvascular filtration rate (115). Although microvascular permeability increases during inflammation, the observed doubling or tripling of the capillary filtration coefficient is not sufficient to explain such a rapid increase in interstitial volume (25). The normal net filtration pressure, i.e., the combined hydrostatic and colloid osmotic pressure gradients, is 0.5–1 mmHg in peripheral tissues (25). The rapid fall in interstitial pressure during the early inflammatory response suggests a 10–100-fold increase in the net filtration pressure. This coincides closely with an early report by Arturson and Mellander in which they calculated an increase in the net filtration pressure following dermal burns of 250–300 mmHg (135). Together, these data strongly suggest that increased negativity of interstitial fluid pressure is the dominant factor in the generation of inflammatory and immune-mediated interstitial edema in loose connective tissue.

## Anti-Edema Mechanisms and Medications

When interstitial edema begins to form, its formation is opposed by a set of mechanisms that act to moderate the magnitude of the interstitial volume increase. These mechanisms are automatic, interdependent and intrinsic to the tissue; however, their effectiveness is not without limit. The result is that the impact of the initial cause of the edema is often muted by the anti-edema mechanisms, but the impact of additional insults can be pronounced. For example, a hypoalbuminemic patient may not display clinically apparent edema, however subsequent administration of intravenous fluids may induce profound edema formation.

Following an increase in the microvascular filtration rate caused by increased venous and microvascular pressures, most organs exhibit four anti-edema responses: (1) an increase in interstitial fluid pressure, (2) a decrease in interstitial COP resulting from protein washdown, (3) an increase in lymph flow, and (4) an increase in trans-serosal flow in organs located within potential spaces. The increase in interstitial fluid pressure and the decrease in COP both act to reduce microvascular filtration according to the relationships modeled in the Starling-Landis equation. The impact of protein washdown as an anti-edema mechanism is blunted by the fact that larger protein molecules washdown to a greater degree than smaller protein species such as albumin. This changes the relationship between protein concentration and COP such that, even at a lower protein concentration (measured as mass per unit volume), there is still a large number of small protein molecules in the interstitial space exerting a considerable COP (136).

Increased lymph flow and serosal transudation provide enhanced removal of interstitial fluid. Because the microlymphatics provide no significant barrier to protein movement (49), lymphatic drainage is more effective than trans-serosal flow at removing protein from the interstitial space; however, this observation is organ dependent. For example, the epicardium is much less permeable to protein than the hepatic serosal surface. Therefore, interstitial protein removal in the heart is more dependent on lymphatic drainage than in the liver (97, 98). This means that serosal transudation in such organs is not independent of lymphatic function. The low serosal protein permeability in organs such as the heart leads to an interesting dynamic in which obstruction of lymphatic drainage causes an increase in interstitial protein concentration and, therefore, a decrease in serosal fluid transudation (29, 97).

Intravenous administration of plasma substitutes containing colloids such as albumin, fresh frozen plasma, dextran or hydroxyethyl starch have been advocated in the treatment of hypovolemia. Their use was encouraged on the supposition that, compared to crystalloid solutions, increases in plasma colloid osmotic pressure would reduce the redistribution of administered fluid to the interstitial space and, thus, maximize the increase in circulating volume. This would improve clinical management of hypovolemia by providing circulatory support while minimizing interstitial edema formation. Unfortunately, several meta-analyses of numerous clinical trials comparing colloid and crystalloid therapy in critically ill humans have failed to demonstrate an improvement in mortality attributable to colloid use (137–139). In addition, the use of some colloids carries an increased risk of negative consequences. Hydroxyethyl starch accumulates in multiple tissues and remains for prolonged periods and, in human trials, its use is correlated with an increased need for renal replacement (140).

These analyses do not provide an explanation for why colloids do not result in an improvement in mortality; however, one reason could be that the fundamental premise supporting their use is flawed. As early as 1987, Michel and Phillips demonstrated that, in single capillaries, lowering of capillary hydrostatic pressure did not lead to steady-state fluid reabsorption into the capillary (141). Rather, as capillary pressure falls, outward filtration falls and then stabilizes at very low, but positive, levels. This coincides with the earlier discussion of the limits of transcapillary refill. When capillary pressure falls, interstitial hydrostatic pressure falls and interstitial COP increases thus preventing steady-state reabsorption. This observation was emphasized recently to point out that steady-state fluid reabsorption does not occur in the vast majority of capillary beds (1). However, another way to look at this phenomenon is that, at the low capillary pressures characteristic of hypovolemia, changes in capillary pressure have little effect on microvascular filtration. This suggests that fluid replacement in hypovolemic patients would not initially increase filtration and, therefore, would not predispose to interstitial edema formation (142). Early in the course of therapy, most of the administered solution would remain in the plasma space regardless of whether it was colloid or crystalloid. An excellent review of the impact of our current understanding of microvascular filtration on intravenous fluid therapy considerations is available (142).

Crystalloid solutions are not considered useful as a treatment for interstitial edema because, in most tissues, the microvascular barrier is freely permeable to sodium and chloride ions. Thus, these ions are unable to generate an osmotic pressure gradient necessary to promote water movement. Therefore, it is surprising that intravenous hypertonic saline appears to be effective in the prevention of experimental hydrostatically-induced intestinal edema (143). In rat experiments using mesenteric venous hypertension and intravenous isotonic saline infusion over a 7-h period to create intestinal edema, the addition of 7.5% intravenous saline solution (4 mL/kg) to the protocol resulted in decreased intestinal wall fluid volume, increased peritoneal and luminal fluid volumes and increased urine output (144). The hydraulic conductivity across the intestinal seromuscular layer was significantly increased in the hypertonic saline group which could explain fluid movement from the interstitium to the peritoneal space. Western blot analysis of the intestinal tissue demonstrated increased expression of aquaporin 4 protein levels in the hypertonic saline group suggesting a possible mechanism for the change in hydraulic conductivity.

## Future Directions

New appreciation of the interstitial space as a site of active regulation and potentially rapid change invites a host of questions regarding clinical impact—questions that may not be answered by current lines of research.

Investigations into the role of the extracellular matrix in the regulation of salt and water balance presently focus on the connection to hypertension. While this is certainly an important issue, particularly for human medicine, it is not the only one. The impact of changes in the skin content of sulfated GAGs on interstitial mechanics is unknown. One could hypothesize that, if an increase in interstitial GAG content induced by a high salt diet were to decrease interstitial compliance, the transcapillary refill that occurs during hemorrhage might be blunted leaving the subject more susceptible to hemorrhagic shock. First, of course, the effect of dietary salt on the extracellular matrix composition needs to be demonstrated in veterinary patients.

The idea that the rapid edema formation associated with inflammation and immune phenomena is caused, in large part, by a fall in interstitial fluid pressure runs counter to previous expectation. The interstitial changes that characterize this process have been studied in skin and tracheal mucosa, in part, because those tissues provide *in vivo* preparations that are stable enough to allow precise measures of interstitial fluid pressure. Loose connective tissue, however, is found throughout the body. It seems likely, therefore, that inflammatory edema formation affecting other tissues, such as the intestines or lung, involves a similar mechanism. It might even be that the lamellar edema that occurs in horses suffering from laminitis occurs initially because of a fall in interstitial fluid pressure in the lamellar dermis.

Published reports offer some hope for finding effective therapies for inflammation-induced edema and shock. PDGF-BB, insulin and α-trinositol have all been shown experimentally to moderate or reverse the fall in interstitial fluid pressure and interstitial edema formation when administered post-treatment, as well as pre-treatment, for inflammatory edema (120, 122, 131, 134). The ability to effectively intervene after the onset of the disease state makes the use of these agents or their analogs more clinically feasible. Studies demonstrating that pancreatic enzyme dilution and inhibition as well as administration of vagal agonists improve outcomes when used to treat circulatory shock-related organ dysfunction also provide new avenues for clinical investigation. Also, since the pathogenesis of inflammatory edema likely overlaps with the pathogenesis of shock-induced intestinal ischemia-reperfusion, there will likely be effective common therapeutic approaches for these conditions.

Human clinical trials suggest a benefit to the use of intravenous hypertonic saline in edematous patients based on it being used as a *replacement* for isotonic solutions (145). This benefit is believed due to the reduced administered volume and the avoidance of fluid overload when compared to isotonic solutions. However, experimental studies in rats demonstrate an ability to reduce intestinal edema formation when hypertonic saline is used *in addition* to isotonic solutions (143, 144, 146). Therefore, the observed benefit in these studies cannot be attributed to a reduction in the volume of intravenous solutions administered. Proposed mechanisms for this additional benefit include aquaporin-mediated changes in tissue permeability leading to fluid shifts as well as modulation of trauma-induced inflammatory and immune processes (144, 147, 148).

## Author Contributions

RS was responsible for all stages of authorship including research and writing.

## Conflict of Interest

The author declares that the research was conducted in the absence of any commercial or financial relationships that could be construed as a potential conflict of interest.
